# The functional connectivity of the middle frontal cortex predicts ketamine’s outcome in major depressive disorder

**DOI:** 10.3389/fnins.2022.956056

**Published:** 2022-09-15

**Authors:** Fan Zhang, Chengyu Wang, Xiaofeng Lan, Weicheng Li, Ling Fu, Yanxiang Ye, Haiyan Liu, Kai Wu, Yanling Zhou, Yuping Ning

**Affiliations:** ^1^The First School of Clinical Medicine, Southern Medical University, Guangzhou, China; ^2^The Affiliated Brain Hospital of Guangzhou Medical University, Guangzhou, China; ^3^Guangdong Engineering Technology Research Center for Translational Medicine of Mental Disorders, Guangzhou, China; ^4^School of Biomedical Sciences and Engineering, South China University of Technology, Guangzho, China

**Keywords:** ketamine, major depressive disorder, degree centrality (DC), functional connectivity, default mode network, biomarker

## Abstract

**Background:**

Ketamine, a robust antidepressant, has promising potential in the treatment of major depressive disorder (MDD). However, it does not work for all MDD patients, and the mechanism underlying its anti-depressive effects is unclear. Researchers have explored the mechanisms of ketamine action in MDD patients through MRI, a technique that measures brain activity intuitively. Notably, many MRI results were inconsistent because they selected different brain regions as seeds, particularly with respect to functional connectivity (FC) analysis. To eliminate the influence of prior seeds as much as possible, we used the significantly different results in degree centrality (DC) analysis as seeds to explore the FC changes in MDD patients to identify an imaging biomarker of ketamine’s effect.

**Methods:**

Forty-four MDD patients and 45 healthy controls (HCs) were included in the study. Patients, aged 18–65, received six intravenous ketamine injections over 12 days. Depressive symptoms were estimated and MRI scans were performed at baseline and the day after the sixth infusion. We estimated FC differences between responders, non-responders and HCs using the region that showed significant differences between responders and non-responders in DC analysis as the seed. The correlation between the MADRS changes and zFC values was performed, and the potential of zFC values to be a neuroimaging biomarker was explored using the receiver operating characteristic curve.

**Result:**

Compared with non-responders, responders had significantly decreased DC values in the right middle frontal gyrus (MFG). In the analysis of FC using the region that showed significant differences in DC as a seed, there was a significant difference in the region of the right supplementary motor area (SMA) among responders, non-responders, and HCs. This region also overlapped with the bilateral median cingulate gyrus. In *post hoc* analysis, responders had higher FC than non-responders and HCs, and non-responders had lower FC than HCs. Importantly, the FC between the MFG and SMA (overlapping bilateral median cingulate gyrus) was correlated with the improvement of symptoms, which was estimated by the Mongomery-Asberg Depression Scale (MADRS). FC has the potential to be an imaging biomarker that can predict the ketamine effect in MDD patients according to the receiver operating characteristic curve analysis.

**Conclusion:**

Our results revealed that FC between the SMG and SMA and mACC was highly correlated with depressive symptoms and has the potential to be a neuroimaging biomarker to predict the effect of ketamine in MDD.

## Introduction

Ketamine, an N-methyl-D-aspartate receptor (NMDAR) antagonist, provides hope for patients with major depressive disorder (MDD) due to its quick and potent antidepressant effects (Serafini et al., 2014). Studies demonstrated that a single intravenous infusion of ketamine (0.5 mg/kg) had an antidepressant effect 40 min post-infusion, and the crest value occurred 1 day after infusion (Berman et al., 2000; Zarate et al., 2006; Murrough et al., 2013a). Previous studies have shown that MDD patients had a prolonged response after receiving a total of 6 ketamine injections (Murrough et al., 2013b; Zheng et al., 2018). Nevertheless, the mechanism of the antidepressant effect of ketamine is not known.

Recently, an increasing number of researchers have used neuroimaging to explore the mechanism of ketamine action in MDD through functional connectivity (FC), a neuroimaging analysis using blood oxygenation level-dependent (BOLD) signals obtained from the *in vivo* brain (Scheidegger et al., 2016; Kraguljac et al., 2017; Teng et al., 2018; Chen et al., 2019; Mkrtchian et al., 2021; Rivas-Grajales et al., 2021). Mkrtchian et al. (2021) revealed that FC between the ventral striatum-left dorsolateral prefrontal cortex, dorsal caudate-right ventrolateral prefrontal cortex, dorsal caudal putamen-pregenual anterior cingulate cortex, and ventral rostral putamen-orbitofrontal cortex increased in treat-resistant depressive participants after ketamine treatment. However, Kraguljac et al. (2017) found that there were no areas that showed increased hippocampus connectivity during a ketamine challenge. Thus, these results are often inconsistent.

These heterogeneous results are due to the different “seeds” in FC analysis, *a priori* brain regions selected based on information obtained from task activation studies, functional neuroanatomy, or even structural deficits (Craddock et al., 2009; Zhang et al., 2016). To avoid this influence, we used the degree centrality (DC) to select the seed (Zuo et al., 2012), which is a kind of network analysis that estimates each node’s correlation with the others to measure the importance of each node (Wang et al., 2011; Yang et al., 2014). This is a measure of the importance of each voxel from a whole-brain network perspective, and getting seeds from it can partly reduce the influence (Cheng et al., 2022). Increased DC values in a brain region mean this region plays a key role in brain activity.

In the present study, we obtained seed from DC analysis to reduce bias based on previous brain regions. Then we estimated the different FC among responders, non-responders, and HCs at baseline. The relationship between Mongomery-Asberg Depression Scale (MADRS) score changes and FC values was investigated. In addition, the receiver operating characteristic curve analysis was used to explore the potential of the zFC values as a neuroimaging biomarker of ketamine’s antidepressant effect in MDD patients. We wish we could find a reliable neuroimaging biomarker to predict the effect of ketamine in MDD patients.

## Patients and method

### Study participants

Participants were recruited from a clinical trial (ChiCTR-OOC-17012239) in the Affiliated Brain Hospital of Guangzhou Medical University. Two experienced psychiatrists used the Diagnostic and Statistical Manual of Mental Disorders-5 (DSM-5, SCID) to screen patients. Our study was approved by the Clinical Research Ethics Committee of the Affiliated Brain Hospital of Guangzhou Medical University.

The inclusion criteria for the MDD group were as follows: (a) aged 18–65, (b) 17-item Hamilton Depression Rating Scale (HAMD-17) score ≥ 17 at baseline, (c) failure of two adequate antidepressant treatments or Beck Scale for Suicide Ideation-Part I score ≥ 2 at baseline.

Healthy controls (HCs) and their family members must have had no DSM-5 diagnosis.

The exclusion criteria for both MDD patients and HCs were as follows: (a) psychotic symptoms; (b) alcohol or substance abuse, (c) any serious or unstable medical conditions at present, or (d) MRI contraindications.

Patients with psychiatric medication treatment, were required to maintain a stable dosage over 4 weeks before ketamine infusion and take stable medications throughout the infusion period.

Forty-four patients were recruited and all signed the consent form. Excluding four patients with maximum head motion parameters over 2 mm or 2°, finally, 40 patients were included in the analysis. Forty-five MRI scans from HCs were also included in the analysis.

### Study design

Forty-four MDD patients received six ketamine infusions in 12 days, they were on days 1, 3, 5, 8, 10, and 12, respectively. After an overnight fast, ketamine (0.5 mg/kg) was diluted in saline and injected intravenously through a pump over 40 min. Depressive symptoms and MRI scans were collected at baseline (1 day before the first infusion), and post-treatment (1 day after the sixth infusion). The detailed study design has been described in our previous studies (Zheng et al., 2018; Zhou et al., 2018a,b).

### Rating scales

Depressive symptoms were estimated using the Montgomery-Asberg Scale (MADRS) and the responders were defined as having an improvement in MADRS scores (ΔMADRS%) ≥ 50%. This was calculated as follows: baseline MADRS score minus posttreatment MADRS score, then divided by the baseline MADRS, and finally multiplied by 100%.

### Acquisition of MRI data

Participants completed fMRI scans at baseline and posttreatment. Participants were required to close their eyes but stay awake during the scans. BOLD signals were collected using a 3.0-T Philips Achieva MRI scanner (Philips, the Netherlands). An eight-channel SENSE head coil was used to record fast field echo (FFE) echo-planar images (EPI), the parameters were as follows: repetition time = 2,000 ms; echo time = 30 ms; flip angle = 90°; 33 slices; matrix = 64 × 64; field-of-view = 220 × 220 × 150 mm^3^; voxel size = 3.44 × 3.44 × 4 mm^3^; gap = 0.6 mm; and the number of signal averages (NSA) = 1. The resting fMRI scan (8 min, 43 s) comprised 240 contiguous volumes.

### Preprocessing of MRI data

The MRI data were preprocessed using the toolbox of data processing and analysis for (resting-state) Brain Imaging (DPABI version 6.0),^1^ running in MATLAB R2019b (The Mathworks, Natick, MA, USA).

We converted the data from the digital imaging and communications in medicine (DICOM) to a standard format (Neuroimaging Informatics Technology Initiative). The first 10 time points were removed to keep the signal stable. The remaining images were corrected using slice timing and realignment to reduce the interval scanning time difference and head motion. Four images were excluded for their maximum head motion parameter of over 2 mm or 2°. The remaining images were normalized to the Montreal Neurological Institute (MNI) using EPI templates. Nuisance signals from 24-parameter head motion profiles, white matter signals, cerebrospinal fluid signals, and global signals were removed using linear regression. Detrending was performed to remove the linear drift. To decrease physiological noise, images were filtered at 0.01–0.08 Hz.

### Degree centrality

After preprocessing, the DC value was calculated using DPABI software. The BOLD signal of each voxel was extracted and collected with every other voxel. The number of correlations, which was over 0.25 (*r* > 0.25), was the DC value (Buckner et al., 2009; Wang et al., 2021). Then the DC values were *z*-transformed to acquire the Z score DC value images. Finally, these images were smoothed using a 6 mm × 6 mm × 6 mm full width at half the maximum Gaussian kernel.

### Functional connectivity

The significantly different clusters in the DC map were used as seeds. The average time series of these regions were separately correlated with the remaining voxels to calculate the FC values and then z-transformed to obtain zFC maps of all MDD patients and HCs.

### Statistical analysis

Demographic characteristics, including educational level, duration of illness, baseline MADRS score, posttreatment MADRS score, and the dose of antidepressant (converted to standard fluoxetine equivalents) were compared between responders and non-responders using the Kruskal–Wallis H test. Body mass index (BMI) between responders and non-responders was analyzed using two-sample *t*-tests, and the gender was compared using the chi-square test. The age data and head motion were analyzed among responders, non-responders and HCs using analysis of variance (ANOVA). All of the above were run on SPSS 25.0 software, and the significance threshold was *p* < 0.05.

DC values were compared between responders and non-responders using a two-sample *t*-test analysis with age, BMI, and head motion as covariates in SPM12. AAL 90 was used as a mask in the analysis.

A one-way ANOVA with age, gender, and head motion as covariates was used in SPM12 to explore zFC differences among responders, non-responders, and HCs. The mean zFC values in the different clusters were extracted to conduct a *post hoc* analysis.

Moreover, we explored the relationship between the mean zFC values of MDD patients and ΔMADRS% using Spearman’s correlation analysis. The mean zFC values of responders and non-responders were extracted separately and assessed the diagnostic efficiency using the receiver operating characteristic curve analysis in GraphPad Prism 5 (GraphPad Software Inc., USA).

## Results

### Demographic characteristics and clinical symptoms

The demographic and clinical results are shown in Table 1. There was no significant difference in gender, educational level, duration of illness, head motion, dose of antidepressant, or baseline MADRS score. However, responders were older and had a higher BMI than non-responders (*p* < 0.05). As expected, the responder group showed a higher posttreatment MADRS score than the non-responder group (*p* < 0.05).

**TABLE 1:** Demographics and clinical characteristics of the MDD and HCs at baseline.

	Responders	Non-responders	HCs	*P*
Subjects	24	16	45	–
Gender (female/male)	15/9	9/7	27/18	0.925
Age (Year, mean ± *SD*)	39.79 ± 11.58	30.69 ± 11.09	31.44 ± 7.98	0.002
Education (year)	12 (9, 15)	13.5 (9, 15)	–	0.594
Duration (month)	60 (24, 153)	42 (8.25, 105)	–	0.345
BMI (mean ± *SD*)	24.23 ± 2.70	21.36 ± 3.24	–	0.004
Baseline MADRS	31.5 (26, 34.75)	33 (23.5, 39.25)	–	0.503
Post-treatment MADRS	8 (4.25, 11)	27 (20, 31.5)	–	0.000
Head motion (FD)	0.048 (0.039, 0.063)	0.044 (0.035, 0.055)	0.052 (0.047, 0.066)	0.05
The dose of antidepressant (convert to standard fluoxetine equivalents)	51 (20, 60)	35 (20, 63.75)	–	0.733

HCs, healthy control.

### Degree centrality analysis

**FIGURE 1:**
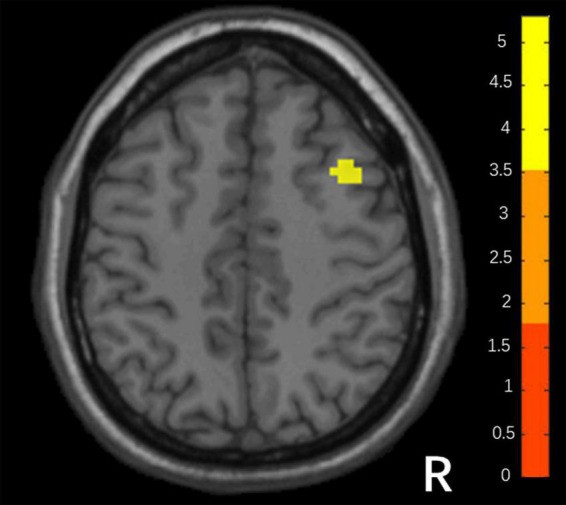
Differences in DC between responders and non-responders. (Two sample *t*-test, voxel- level *p* < 0.001, peak *p* < 0.05 corrected by FDR).

Compared with responders, non-responders had higher values in the DC map (voxel-level *p* < 0.001; peak-level *p* < 0.05 corrected by FDR). It is a cluster located in the right middle frontal gyrus (MFG; *x* = 36, *y* = 15, *z* = 45, *k* = 32), shown in Figure 1.

### Functional connectivity analysis

**FIGURE 2:**
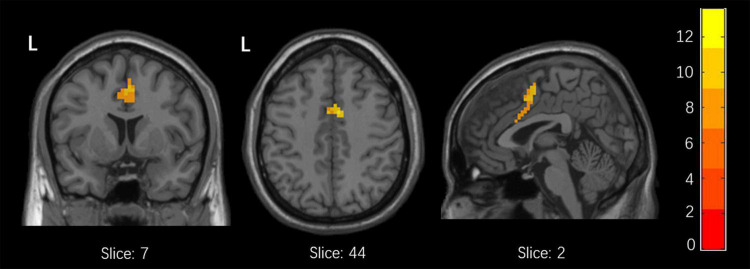
Differences in xFC among responders, non-responders and HCs. (one-Way ANOVA, voxel-level *p* < 0.001, cluster-level *p* < 0.05 corrected by FDR).

**FIGURE 3:**
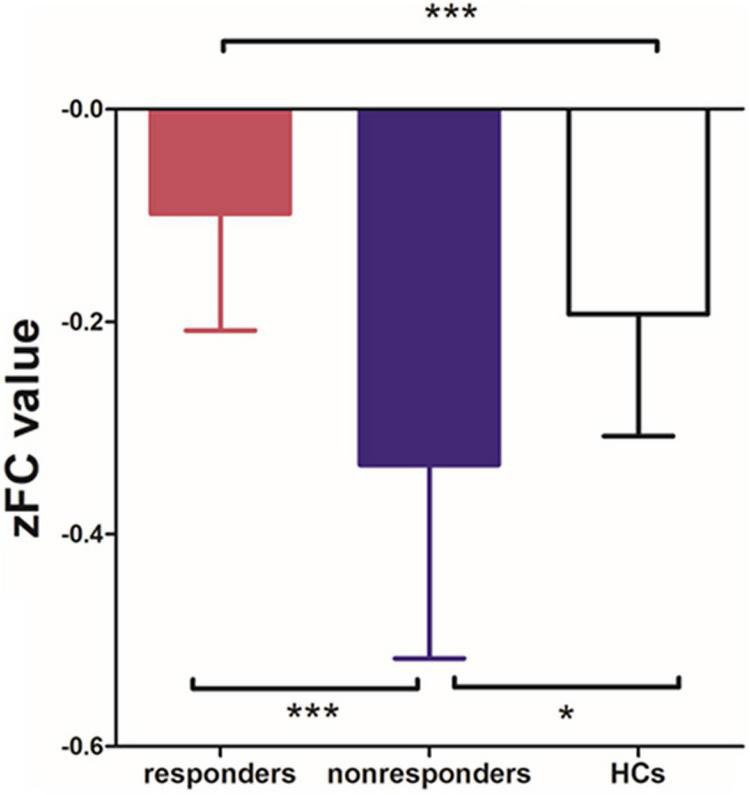
Differences in zFC values among responders, non-responders and HCs. (one-way ANOVA, Bonferroni’s Multiple Comparison Test. *p < 0.05, ***p < 0.0001).

Among the 3 groups, MFG-related zFC maps were different in a region centered in the right supplementary motor area (SMA) (*x* = 6, *y* = 3, *z* = 45, *k* = 90; voxel-level *P* < 0.001, cluster-level *P* < 0.05 corrected by FDR). This region also contained parts of the bilateral median cingulate gyrus (SMA and mACC), as shown in Figure 2. The *post hoc* analysis revealed that responders had higher zFC values than non-responders and HCs, and non-responders had lower zFC values than HCs (Figure 3).

### Relationship between the zFC value and ΔMADRS%

**FIGURE 4:**
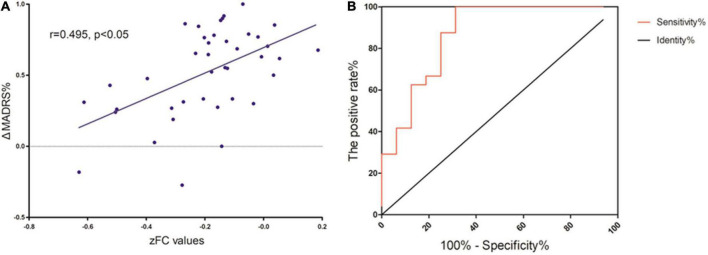
**(A)** Correlation between zFC values and L’lMADRS% in MOD patients. **(B)** ROC curve showing an area under the curve (AUC) of 0.8672 (*p* < 0.001) for the zFC values, with a sensitivity of 100%, and specificity of 68.75%.

As Figure 4 shows, Spearman’s correlation analysis revealed that the zFC values between MFG and SMA and mACC were positively correlated with ΔMADRS% in MDD patients (*r* = 0.495, *P* < 0.05). Importantly, the zFC value has the potential to be a predictor of the effect of ketamine (AUC = 0.872, *P* < 0.001).

## Discussion

In this study, we used the seed obtained from the DC map to explore the FC alterations in patients with MDD. The results revealed that the non-responder group had increased DC compared with the responder group in the region of the right MFG, a part of the DMN. The seed-based FC analysis showed a significant difference in the SMA and mACC among responders, non-responders, and HCs. The zFC in this region had a highly sensitive response to ketamine in MDD after six infusions. It can be used as a neuroimaging biomarker.

The MFG was a major part of the default mode network (DMN). The DMN is a key network in MDD and plays the role of cognitive control and integrating information (Yeshurun et al., 2021; Liu et al., 2022; Pang et al., 2022b). According to the prevalent triple network model, the symptoms of depression can be explained by dysfunction between the DMN, salience network, and central executive network, especially the increased FC in the DMN (Hamilton et al., 2015; Kaiser et al., 2015; Li et al., 2022). Using graph theory-based methods in the data of 821 MDD and 765 HCs, Yang et al. (2021) revealed that patients with MDD were characterized by decreased nodal efficiency in the DMN. Another study compared 848 MDD patients with 794 HCs and also found decreased FC in the DMN (Yan et al., 2019). Our results went one step further and revealed a difference within the MDD group. Liang et al. found two MDD subgroups with differing FC profiles of the DMN from 690 MDD patients; one group exhibited increases in connectivity, and the other subgroup showed decreases in connectivity (Liang et al., 2020). Price et al. (2017) also revealed two subgroups in MDD patients by assessing the difference in connectivity patterns across DMN nodes. A prior study found that ketamine can reduce FC (Price et al., 2017). Moreover, research has found that changes in FC at baseline could predict the effect of ECT (Pang et al., 2022a). In our results, the FC of responders will be toward HCs while non-responders will be far away from HCs if the FC has been reduced. It seems that the two groups qualitatively had different FC in the DMN, although the FC was negative in both groups. This finding supports the theory mentioned above: there were two subgroups in MDD, one of which is sensitive to ketamine while the other is not.

An increasing number of studies have partly revealed the function of the SMA. Gabbay et al. (2013) revealed that anhedonia scores were positively correlated with the intrinsic FC strength of the SMA; Westlund Schreiner et al. (2017) revealed a hyperconnectivity between the amygdala in self-injured adolescents without suicidal, which may show that negative effects have an important link with habitual behaviors; while other researchers showed that the FC value of the right SMA was negatively correlated with depressive symptoms in depressed patients with irritable bowel syndrome (Li et al., 2021). There were also some results regarding the cortical thickness in the SMA. Some studies have shown depressed patients had significantly smaller volumes of the right pre-SMA than control subjects (Exner et al., 2009; Cheng et al., 2010; Gabbay et al., 2013; Westlund Schreiner et al., 2017). Salomons et al. (2012) revealed fractional anisotropy of connected white matter tracts along the corticospinal tract were associated with helplessness and mediated the relationship between the SMA cortical thickness and helplessness. Besteher et al. (2017) revealed that somatization symptoms showed a negative correlation with the gray matter volume of the right SMA. Moreover, Li et al. (2015) found that regional cerebral glucose metabolism (rCMglu) in the bilateral SMA was decreased in the medication-resistant depression (MRD) group than in the non-MRD group, the MRD group patients also had decreased rCMglu in the SMA than the control group, while Chen et al. (2018) revealed that TRD patients who received the 0.5 mg/kg ketamine infusion had significantly higher glucose metabolism in the SMA than those who received the 0.2 mg/kg ketamine infusion, these authors suggest that the persistent antidepressant effect of a 0.5 mg/kg ketamine infusion may be mediated by increased activation in the SMA. These results indicate that the SMA has a tighter connection with MDD and plays an important role in habitual behaviors, depressive symptoms, and helpless feelings.

It’s worth noting that the significant cluster located in the right SMA also overlapped with the bilateral median cingulate gyrus. The cingulate participates in the control of cognition and emotion, and executive attention (Botvinick et al., 2004; Etkin et al., 2006). Recently, an increasing number of studies have shown that the cingulate plays a key role in MDD and bipolar disorder (BP). A large meta-analysis, including 148 MDD patients and 7,957 HCs, indicated that patients with MDD had a thinner cortical anterior cingulate cortex and posterior cingulate cortex (Schmaal et al., 2017). Several studies indicated that the volume of the anterior cingulate cortex was associated with BP, the volume of the anterior midcingulate cortex at baseline was associated with greater symptom improvement after follow-up and patients who remitted had less volume decline than non-remitted patients in the left anterior cingulate cortex during a 3-year follow-up period (Lochhead et al., 2004; McDonald et al., 2004; Frodl et al., 2008; Phillips et al., 2015). Regarding FC, Greicius et al. (2007) found that the FC of the subgenual anterior cingulate cortex was correlated with the depressive episode length. A study using rTMS to treat treatment-resistant depression used sgACC-DLPFC and rACC-IPL connectivity as features, and found responder-non-responder classification accuracies of 84 and 76% (end-of-treatment), 88 and 81% (3-month follow-up) (Ge et al., 2020). Enhanced FC between the right middle cingulum and right medial prefrontal cortex was positively correlated with the duration of depression since onset (Marazzi et al., 2021). This region is a key node in MDD that we cannot ignore.

Our findings should be considered with some limitations. First, patients took antidepressants and received ketamine injections at the same time. Although there was no difference in the dose, it may have affected the FC in the whole brain, thereby, impacting our final result. However, in the present study, it was closer to a real situation in patients’ daily lives. Second, we only explored the FC values in the cerebrum (using the mask of AAL90), excluding the cerebellum. This may cause us to ignore the role of the cerebellum in brain activity. Third, we did not include the baseline MADRS score as a covariate because we did not collect it from HCs. It may improve results in our future studies. Finally, the MDD patients in our study include treatment-resistant depressive patients and patients with suicidal ideation. This could have biased our results.

Our results partially support the DMN’s key role of the DMN in MDD and MDD patients could be identified as two subgroups by FC in the DMN. We also revealed that FC between the DMN and SMA and mACC was more highly correlated with depressive symptoms. In addition, FC has the potential to be a neuroimaging biomarker to predict the ketamine effect.

## Data availability statement

The raw data supporting the conclusions of this article will be made available by the authors, without undue reservation.

## Ethics statement

This study was approved and reviewed by the Clinical Research Ethics Committee of the Affiliated Brain Hospital of Guangzhou Medical University. Written informed consent was obtained from the individual(s) for the publication of any potentially identifiable images or data included in this article.

## Author contributions

FZ: investigation, formal analysis, writing—original drafting, and visualization. YZ: validation, project administration, methodology, and investigation. CW and XL: validation, methodology, and investigation. WL, LF, YY, and HL: investigation. KW: conceptualization. YN: conceptualization, supervision, writing—review and editing. All authors contributed to the article and approved the submitted version.

## Footnotes

1
http://rfmri.org/DPARSF

## References

[B1] Berman R. M. Cappiello A. Anand A. Oren D. A. Heninger G. R. Charney D. S. (2000). Antidepressant effects of ketamine in depressed patients. *Biol. Psychiatry* 47 351–354. 10.1016/S0006-3223(99)00230-9 10686270

[B2] Besteher B. Gaser C. Langbein K. Dietzek M. Sauer H. Nenadić I. (2017). Effects of subclinical depression, anxiety and somatization on brain structure in healthy subjects. *J. Affect. Disord.* 215 111–117. 10.1016/j.jad.2017.03.039 28319687

[B3] Botvinick M. M. Cohen J. D. Carter C. S. (2004). Conflict monitoring and anterior cingulate cortex: An update. *Trends Cogn. Sci.* 8 539–546. 10.1016/j.tics.2004.10.003 15556023

[B4] Buckner R. L. Sepulcre J. Talukdar T. Krienen F. M. Liu H. Hedden T. (2009). Cortical hubs revealed by intrinsic functional connectivity: Mapping, assessment of stability, and relation to Alzheimer’s disease. *J. Neurosci.* 29 1860–1873. 10.1523/JNEUROSCI.5062-08.2009 19211893 PMC2750039

[B5] Chen M. Li C. Lin W. Hong C. Tu P. Bai Y. (2018). Persistent antidepressant effect of low-dose ketamine and activation in the supplementary motor area and anterior cingulate cortex in treatment-resistant depression: A randomized control study. *J. Affect. Disord.* 225 709–714. 10.1016/j.jad.2017.09.008 28922734

[B6] Chen M. H. Lin W. C. Tu P. C. Li C. T. Bai Y. M. Tsai S. J. (2019). Antidepressant and antisuicidal effects of ketamine on the functional connectivity of prefrontal cortex-related circuits in treatment-resistant depression: A double-blind, placebo-controlled, randomized, longitudinal resting fMRI study. *J. Affect. Disord.* 259 15–20. 10.1016/j.jad.2019.08.022 31437695

[B7] Cheng B. Roberts N. Zhou Y. Wang X. Li Y. Chen Y. (2022). Social support mediates the influence of cerebellum functional connectivity strength on postpartum depression and postpartum depression with anxiety. *Transl. Psychiatry* 12:54. 10.1038/s41398-022-01781-9 35136017 PMC8826948

[B8] Cheng Y. Xu J. Chai P. Li H. Luo C. Yang T. (2010). Brain volume alteration and the correlations with the clinical characteristics in drug-naïve first-episode MDD patients: A voxel-based morphometry study. *Neurosci. Lett.* 480 30–34. 10.1016/j.neulet.2010.05.075 20594947

[B9] Craddock R. C. Holtzheimer P. R. Hu X. P. Mayberg H. S. (2009). Disease state prediction from resting state functional connectivity. *Magn. Reson. Med.* 62 1619–1628. 10.1002/mrm.22159 19859933 PMC3749911

[B10] Etkin A. Egner T. Peraza D. M. Kandel E. R. Hirsch J. (2006). Resolving Emotional Conflict: A Role for the Rostral Anterior Cingulate Cortex in Modulating Activity in the Amygdala. *Neuron* 51 871–882. 10.1016/j.neuron.2006.07.029 16982430

[B11] Exner C. Lange C. Irle E. (2009). Impaired implicit learning and reduced pre-supplementary motor cortex size in early-onset major depression with melancholic features. *J. Affect. Disord.* 119 156–162. 10.1016/j.jad.2009.03.015 19345999

[B12] Frodl T. S. Koutsouleris N. Bottlender R. Born C. Jäger M. Scupin I. (2008). Depression-related variation in brain morphology over 3 years: Effects of stress? *Arch. Gen. Psychiatry* 65 1156–1165. 10.1001/archpsyc.65.10.1156 18838632

[B13] Gabbay V. Ely B. A. Li Q. Bangaru S. D. Panzer A. M. Alonso C. M. (2013). Striatum-Based Circuitry of Adolescent Depression and Anhedonia. *J. Am. Acad. Child Adolesc. Psychiatr.* 52 628–641. 10.1016/j.jaac.2013.04.003 23702452 PMC3762469

[B14] Ge R. Downar J. Blumberger D. M. Daskalakis Z. J. Vila-Rodriguez F. (2020). Functional connectivity of the anterior cingulate cortex predicts treatment outcome for rTMS in treatment-resistant depression at 3-month follow-up. *Brain Stimul.* 13 206–214. 10.1016/j.brs.2019.10.012 31668646

[B15] Greicius M. D. Flores B. H. Menon V. Glover G. H. Solvason H. B. Kenna H. (2007). Resting-state functional connectivity in major depression: Abnormally increased contributions from subgenual cingulate cortex and thalamus. *Biol. Psychiatr.* 62 429–437. 10.1016/j.biopsych.2006.09.020 17210143 PMC2001244

[B16] Hamilton J. P. Farmer M. Fogelman P. Gotlib I. H. (2015). Depressive Rumination, the Default-Mode Network, and the Dark Matter of Clinical Neuroscience. *Biol. Psychiatry* 78 224–230. 10.1016/j.biopsych.2015.02.020 25861700 PMC4524294

[B17] Kaiser R. H. Andrews-Hanna J. R. Wager T. D. Pizzagalli D. A. (2015). Large-Scale Network Dysfunction in Major Depressive Disorder: A Meta-analysis of Resting-State Functional Connectivity. *JAMA Psychiat.* 72 603–611. 10.1001/jamapsychiatry.2015.0071 25785575 PMC4456260

[B18] Kraguljac N. V. Frolich M. A. Tran S. White D. M. Nichols N. Barton-McArdle A. (2017). Ketamine modulates hippocampal neurochemistry and functional connectivity: A combined magnetic resonance spectroscopy and resting-state fMRI study in healthy volunteers. *Mol. Psychiatry* 22 562–569. 10.1038/mp.2016.122 27480494 PMC5562151

[B19] Li C. Su T. Wang S. Tu P. Hsieh J. (2015). Prefrontal glucose metabolism in medication-resistant major depression. *Brit. J. Psychiat.* 206 316–323. 10.1192/bjp.bp.113.140434 25657357

[B20] Li J. He P. Lu X. Guo Y. Liu M. Li G. (2021). A Resting-state Functional Magnetic Resonance Imaging Study of Whole-brain Functional Connectivity of Voxel Levels in Patients With Irritable Bowel Syndrome With Depressive Symptoms. *J. Neurogastroenterol.* 27 248–256. 10.5056/jnm20209 33795543 PMC8026363

[B21] Li Y. Li Y. Wei Q. Bai T. Wang K. Wang J. (2022). Mapping intrinsic functional network topological architecture in major depression disorder after electroconvulsive therapy. *J. Affect. Disord.* 311 103–109. 10.1016/j.jad.2022.05.067 35594966

[B22] Liang S. Deng W. Li X. Greenshaw A. J. Wang Q. Li M. (2020). Biotypes of major depressive disorder: Neuroimaging evidence from resting-state default mode network patterns. *Neuroimage Clin.* 28:102514. 10.1016/j.nicl.2020.102514 33396001 PMC7724374

[B23] Liu C. Han T. Xu Z. Liu J. Zhang M. Du J. (2022). Modulating Gamma Oscillations Promotes Brain Connectivity to Improve Cognitive Impairment. *Cereb. Cortex* 32 2644–2656. 10.1093/cercor/bhab371 34751749

[B24] Lochhead R. A. Parsey R. V. Oquendo M. A. Mann J. J. (2004). Regional brain gray matter volume differences in patients with bipolar disorder as assessed by optimized voxel-based morphometry. *Biol. Psychiat.* 55 1154–1162. 10.1016/j.biopsych.2004.02.026 15184034

[B25] Marazzi S. Kiper P. Palmer K. Agostini M. Turolla A. (2021). Effects of vibratory stimulation on balance and gait in Parkinson’s disease: A systematic review and meta-analysis. *Eur. J. Phys. Rehab. Med.* 57 254–264. 10.23736/S1973-9087.20.06099-2 31939269

[B26] McDonald C. Bullmore E. T. Sham P. C. Chitnis X. Wickham H. Bramon E. (2004). Association of genetic risks for schizophrenia and bipolar disorder with specific and generic brain structural endophenotypes. *Arch. Gen. Psychiatr.* 61 974–984. 10.1001/archpsyc.61.10.974 15466670

[B27] Mkrtchian A. Evans J. W. Kraus C. Yuan P. Kadriu B. Nugent A. C. (2021). Ketamine modulates fronto-striatal circuitry in depressed and healthy individuals. *Mol. Psychiatr.* 26 3292–3301. 10.1038/s41380-020-00878-1 32929215 PMC8462973

[B28] Murrough J. W. Iosifescu D. V. Chang L. C. Al J. R. Green C. E. Perez A. M. (2013a). Antidepressant efficacy of ketamine in treatment-resistant major depression: A two-site randomized controlled trial. *Am. J. Psychiatr.* 170 1134–1142. 10.1176/appi.ajp.2013.13030392 23982301 PMC3992936

[B29] Murrough J. W. Perez A. M. Pillemer S. Stern J. Parides M. K. Aan H. R. M. (2013b). Rapid and longer-term antidepressant effects of repeated ketamine infusions in treatment-resistant major depression. *Biol. Psychiatr.* 74 250–256. 10.1016/j.biopsych.2012.06.022 22840761 PMC3725185

[B30] Pang Y. Wei Q. Zhao S. Li N. Li Z. Lu F. (2022a). Enhanced default mode network functional connectivity links with electroconvulsive therapy response in major depressive disorder. *J. Affect. Disord.* 306 47–54. 10.1016/j.jad.2022.03.035 35304230

[B31] Pang Y. Zhao S. Li Z. Li N. Yu J. Zhang R. (2022b). Enduring effect of abuse: Childhood maltreatment links to altered theory of mind network among adults. *Hum. Brain Mapp.* 43 2276–2288. 10.1002/hbm.25787 35089635 PMC8996351

[B32] Phillips J. L. Batten L. A. Tremblay P. Aldosary F. Blier P. (2015). A Prospective, Longitudinal Study of the Effect of Remission on Cortical Thickness and Hippocampal Volume in Patients with Treatment-Resistant Depression. *Int. J. Neuropsychoph.* 18:v37. 10.1093/ijnp/pyv037 25829180 PMC4571636

[B33] Price R. B. Gates K. Kraynak T. E. Thase M. E. Siegle G. J. (2017). Data-Driven Subgroups in Depression Derived from Directed Functional Connectivity Paths at Rest. *Neuropsychopharmacology* 42 2623–2632. 10.1038/npp.2017.97 28497802 PMC5686504

[B34] Rivas-Grajales A. M. Salas R. Robinson M. E. Qi K. Murrough J. W. Mathew S. J. (2021). Habenula Connectivity and Intravenous Ketamine in Treatment-Resistant Depression. *Int. J. Neuropsychopharmacol.* 24 383–391. 10.1093/ijnp/pyaa089 33249434 PMC8130203

[B35] Salomons T. V. Moayedi M. Weissman-Fogel I. Goldberg M. B. Freeman B. V. Tenenbaum H. C. (2012). Perceived helplessness is associated with individual differences in the central motor output system. *Eur. J. Neurosci.* 35 1481–1487. 10.1111/j.1460-9568.2012.08048.x 22564074

[B36] Scheidegger M. Henning A. Walter M. Lehmann M. Kraehenmann R. Boeker H. (2016). Ketamine administration reduces amygdalo-hippocampal reactivity to emotional stimulation. *Hum. Brain Mapp.* 37 1941–1952. 10.1002/hbm.23148 26915535 PMC6867525

[B37] Schmaal L. Hibar D. P. Samann P. G. Hall G. B. Baune B. T. Jahanshad N. (2017). Cortical abnormalities in adults and adolescents with major depression based on brain scans from 20 cohorts worldwide in the ENIGMA Major Depressive Disorder Working Group. *Mol. Psychiatry* 22 900–909. 10.1038/mp.2016.60 27137745 PMC5444023

[B38] Serafini G. Howland R. H. Rovedi F. Girardi P. Amore M. (2014). The role of ketamine in treatment-resistant depression: A systematic review. *Curr. Neuropharmacol.* 12 444–461. 10.2174/1570159X12666140619204251 25426012 PMC4243034

[B39] Teng C. Zhou J. Ma H. Tan Y. Wu X. Guan C. (2018). Abnormal resting state activity of left middle occipital gyrus and its functional connectivity in female patients with major depressive disorder. *BMC Psychiatry* 18:370. 10.1186/s12888-018-1955-9 30477561 PMC6258168

[B40] Wang J. H. Zuo X. N. Gohel S. Milham M. P. Biswal B. B. He Y. (2011). Graph theoretical analysis of functional brain networks: Test-retest evaluation on short- and long-term resting-state functional MRI data. *PLoS One* 6:e21976. 10.1371/journal.pone.0021976 21818285 PMC3139595

[B41] Wang L. Hu F. Wang W. Li Q. Li Y. Zhu J. (2021). Altered brain intrinsic functional hubs and connectivity associated with relapse risk in heroin dependents undergoing methadone maintenance treatment: A resting-state fMRI study. *Drug Alcohol Depend.* 219:108503. 10.1016/j.drugalcdep.2020.108503 33444899

[B42] Westlund Schreiner M. Klimes-Dougan B. Mueller B. A. Eberly L. E. Reigstad K. M. Carstedt P. A. (2017). Multi-modal neuroimaging of adolescents with non-suicidal self-injury: Amygdala functional connectivity. *J. Affect. Disord.* 221 47–55. 10.1016/j.jad.2017.06.004 28628767 PMC5555154

[B43] Yan C. G. Chen X. Li L. Castellanos F. X. Bai T. J. Bo Q. J. (2019). Reduced default mode network functional connectivity in patients with recurrent major depressive disorder. *Proc. Natl. Acad. Sci. U.S.A.* 116 9078–9083. 30979801 10.1073/pnas.1900390116 PMC6500168

[B44] Yang H. Chen X. Chen Z. B. Li L. Li X. Y. Castellanos F. X. (2021). Disrupted intrinsic functional brain topology in patients with major depressive disorder. *Mol. Psychiatry* 26 7363–7371. 10.1038/s41380-021-01247-2 34385597 PMC8873016

[B45] Yang Y. Dong Y. Chawla N. V. (2014). Predicting node degree centrality with the node prominence profile. *Sci. Rep.* 4:7236. 10.1038/srep07236 25429797 PMC4246206

[B46] Yeshurun Y. Nguyen M. Hasson U. (2021). The default mode network: Where the idiosyncratic self meets the shared social world. *Nat. Rev. Neurosci.* 22 181–192. 10.1038/s41583-020-00420-w 33483717 PMC7959111

[B47] Zarate C. J. Singh J. B. Carlson P. J. Brutsche N. E. Ameli R. Luckenbaugh D. A. (2006). A randomized trial of an N-methyl-D-aspartate antagonist in treatment-resistant major depression. *Arch. Gen. Psychiatry* 63 856–864. 10.1001/archpsyc.63.8.856 16894061

[B48] Zhang B. Li M. Qin W. Demenescu L. R. Metzger C. D. Bogerts B. (2016). Altered functional connectivity density in major depressive disorder at rest. *Eur. Arch. Psychiatry Clin. Neurosci.* 266 239–248. 10.1007/s00406-015-0614-0 26265034

[B49] Zheng W. Zhou Y. Liu W. Wang C. Zhan Y. Li H. (2018). Rapid and longer-term antidepressant effects of repeated-dose intravenous ketamine for patients with unipolar and bipolar depression. *J. Psychiatr. Res.* 106 61–68. 10.1016/j.jpsychires.2018.09.013 30278319

[B50] Zhou Y. Zheng W. Liu W. Wang C. Zhan Y. Li H. (2018a). Antidepressant effect of repeated ketamine administration on kynurenine pathway metabolites in patients with unipolar and bipolar depression. *Brain Behav. Immun.* 74 205–212. 10.1016/j.bbi.2018.09.007 30213652

[B51] Zhou Y. Zheng W. Liu W. Wang C. Zhan Y. Li H. (2018b). Neurocognitive effects of six ketamine infusions and the association with antidepressant response in patients with unipolar and bipolar depression. *J. Psychopharmacol.* 32 1118–1126. 10.1177/0269881118798614 30260273

[B52] Zuo X. N. Ehmke R. Mennes M. Imperati D. Castellanos F. X. Sporns O. (2012). Network centrality in the human functional connectome. *Cereb. Cortex* 22 1862–1875. 10.1093/cercor/bhr269 21968567

